# Enjoyment and Intensity of Physical Activity in Immersive Virtual Reality Performed on Innovative Training Devices in Compliance with Recommendations for Health

**DOI:** 10.3390/ijerph16193673

**Published:** 2019-09-30

**Authors:** Małgorzata Dębska, Jacek Polechoński, Arkadiusz Mynarski, Piotr Polechoński

**Affiliations:** 1 Institute of Sport Sciences, The Jerzy Kukuczka Academy of Physical Education in Katowice, 43-512 Katowice, Poland; m.debska@awf.katowice.pl (M.D.); a.mynarski@awf.katowice.pl (A.M.); 2 Faculty of Physiotherapy, The Jerzy Kukuczka Academy of Physical Education in Katowice, 43-512 Katowice, Poland; piotr.polechonski.kontakt@gmail.com

**Keywords:** health-oriented physical activity, immersive virtual reality, virtual reality, intensity of physical activity

## Abstract

The aim of the study is to assess the enjoyment and intensity of physical exercise while practicing physical activity (PA) in immersive virtual reality (IVR) using innovative training devices (omni-directional Omni treadmill and Icaros Pro flight simulator). The study also contains the results of subjective research on the usefulness of such a form of PA in the opinion of users. In total, 61 adults (10 women and 51 men) took part in the study. To assess the enjoyment level (EL) Interest/Enjoyment subscale of Intrinsic Motivation Inventory (IMI) was used. Exercise intensity was assessed during 10-min sessions of active video games (AVGs) in IVR based on heart rate (HR). The average enjoyment level during physical exercise in IVR on the tested training devices was high (Omni 5.74 points, Icaros 5.60 points on a 1–7 Likert scale) and differed significantly in favor of PA on Omni. In the opinion of the majority of participants, AVGs in IVR on the tested devices constitute a sufficiently useful form of PA to meet the needs of leisure time activities, and they can even replace some forms of physical effort performed in a classic way. The intensity of PA during games on training devices was at the level recommended for health benefits for 80.55% (Omni) and 50.77% (Icaros Pro) of its duration. Based on the conducted research, it can be assumed that AVGs in IVR using a multi-directional treadmill and a flight simulator can be an effective tool for increasing participation in health-oriented PA.

## 1. Introduction

The lack of movement (hypokinesia) is the main cause of the incidence of chronic non-communicable diseases, causing 71% of all deaths per year, including as much as 40% of premature deaths [1]. Therefore, health promotion activities are currently focused on searching for tools popularizing modern physical activity (PA), attractive for people, tailored to their interests, fitness abilities, and leisure time budgets.

The development of modern technology is considered to be one of the reasons for the decreasing PA level in everyday life [2]. Due to the fact that they constitute an inseparable element of the development of civilization in recent years, technological innovations have been used to popularize PA among the public. The first manifestation of this action was the creation of active video games (AVGs) in which the player controls the course of the game with movements of his/her body, becoming its active participant and not a passive player. Subsequently, the so-called “Exergames” were introduced, i.e., differentiated interactive training programs, often with the assistance of a virtual trainer, the goal of which is PA.

Another aspect of using technological progress in improving public health is the transfer of AVG to virtual reality (VR)—a computer-created space to which users move using special goggles. These goggles enable the players to be cut off from the real world, making them part of the game and multiplying the emotions they experience during it [3]. This most advanced technique, enabling the highest degree of realism in which a person is cut off from the visual and auditory stimuli of the real environment, and instead receives the image and sound, and even tactile sensations of the simulated world, is often referred to as immersive virtual reality (IVR) [4,5].

Due to the growing demand for VR devices in various life areas [6], also for the medical and fitness industry, special training devices for training in VR began to be created, e.g., Omni-directional treadmills, flight and diving simulators, cycling simulators etc., are created. These training devices are equipped with sensors that reflect the user’s body movements in VR. Thanks to the devices for PA in VR, the users becomes a part of the artificially created world, and the movements of their body control the course of a chosen game, sports training or visiting virtual destinations [3,7].

So far, published scientific reports confirm a high level of enjoyment during various AVGs and exergames [8,9,10]. It should be noted that according to the results of research on components of behavioral engagement in performing PA, enjoyment is its significant predictor, regardless of age of the study participants [11,12,13,14] and their health status [15,16,17]. In addition, several publications have shown that the attractiveness of various forms of PA in VR is higher in relation to identical forms of PA undertaken in the classical way [3,18,19]. It was also observed that due to the high rating of the attractiveness of games of this type, players are able to perform PA longer in an interactive form, compared to classic PA, which may translate into better health effects [20].

The research interest in verifying the level of physical activity in VR increased. The results of monitoring the parameters of physical exercise during many active video games showed that they are at the level recommended for health by international organizations [21,22,23,24,25,26] and contribute to the health benefits of both healthy people [20,27,28] and patients [29,30,31]. At the same time, it was noticed that the intensity and, consequently, the caloric cost of PA in VR is very diverse, depending on the form of movement, level of involvement of the muscle apparatus (limb vs. the whole body), level of difficulty of the game, experience in playing [23,32,33]. Therefore, it seems important to verify the physiological response to physical efforts undertaken during such kind of PA to select those with a pro-health character.

Analysis of the few research studies concerning PA in VR that have been created over the last few years, indicates the possibilities of using this modern technology in physiotherapy [22,31,34]. A study conducted by Baños et al. [18] shows that walking on a treadmill performed in the virtual world can be better tolerated by obese children than the same physical effort in a traditional form. VR allows for the distraction of the participants from the discomfort that accompanies PA. According to the authors, the use of VR technology in training programs may be a factor increasing motivation to exercise, which may be important in the prevention of overweight and in the fight against obesity. Also a study conducted by Matsangidou et al. [19] demonstrated that PA in VR can affect the perception of pain observed during isometric exercise, what results in lower subjective perception of exercise severity and, consequently, the possibility of continuing the activity and a higher assessment of its attractiveness in comparison with PA performed in a classical manner. Therefore PA in VR can be also used in therapeutic treatment in patients with other dysfunctions, which are accompanied by various ailments associated with physical effort [18,19,29,34,35,36].

Previously published articles on PA in VR focus on the possibility of its implementation in public health activities. Many publications emphasize the potential of this form of movement, recognizing it as a modern trend in healthcare [37,38], mainly in the field of secondary prevention [18,34,35]. There are also reports regarding the assessment of gait parameters in a virtual environment [39,40]. However, there are no scientific reports verifying the level of enjoyment and parameters of physical exercise in the context of pro-health recommendations, during such a form of PA in the case of people without a diagnosed disease, which is the basis for assessing its suitability in the universal health promotion and prevention of. Moreover, in the literature, there are currently no publications presenting the results of research on this issue with the use of special training devices for PA in VR. Meanwhile, due to the high level of enjoyment during AVGs in VR and health-oriented character of many of them, such a form of PA can activate classic computer games enthusiasts, among who the phenomenon of hypokinesia occurs quite often. Performing exercises with greater enjoyment can also increase the frequency of their undertaking, which is commonly insufficient for obtaining health benefits. A new, attractive form of movement, which can be taken regardless of weather conditions, can complement the training of regularly physically active people looking for an alternative to traditional PA forms.

In connection with the above, the aim of the study was to evaluate enjoyment and intensity of physical effort while practicing physical activity in immersive VR using innovative training devices (omni-directional Omni treadmill and Icaros Pro flight simulator). The study also contains the results of subjective research on the usefulness of such a form of physical effort in the opinion of users.

## 2. Materials and Methods

### 2.1. Participants

Sixty-one adults participated in the study They were recruited from among participants of the Silesian Festival of Science (Poland), where equipment and software allowing for PA in VR was presented. From all participants of the above-mentioned festival, people meeting the inclusion criteria (age >18 years, signing a statement about good general health and the lack of medical contraindications to participate in the study or physical limitations affecting exercise, e.g., pregnancy, injury, et al., no history of seizures or epilepsy, taking any medications affecting heart rate) were invited to participate in the study. A schedule of tests was arranged and people who joined them and completed their full program were allocated to the intervention group. In the training programme using the Omni treadmill (Virtuix Inc., Austin, TX, USA) thirty-six adults participated, including six women (age 28.33 ± 11.60 years, height 168.17 ± 5.85 cm, weight 61.33 ± 5.57 kg) and thirty men (age 25.57 ± 8.23 years, height 179.20 ± 5.91 cm, weight 79.95 ± 10.20 kg). Icaros Pro flight simulator (Icaros GmbH, Martinsried, Germany) was tested by twenty-five people: four women (age 32.00 ± 13.04 years, height 165.50 ± 5.87 cm, weight 61.00 ± 7.16 kg) and twenty-one men (age 24.71 ± 8.65 years, height 178.38 ± 7.25 cm, weight 77.74 ± 10.49 kg). Among all the studied people, eleven young men (age 19.73 ± 2.49 years, height 176.27 ± 6.34 cm, weight 74.05 ± 9.86 kg) completed training sessions on both training devices, therefore the comparison of intensity of physical activity and the enjoyment level during PA on selected training devices applied to this part of the group only. 

More than half of the participants (34 people) declared that they had previously experienced immersive VR using computer games using VR headset, while no one had previously had the opportunity to practice PA in virtual reality on the tested training devices.

### 2.2. Procedures

**Figure 1:**
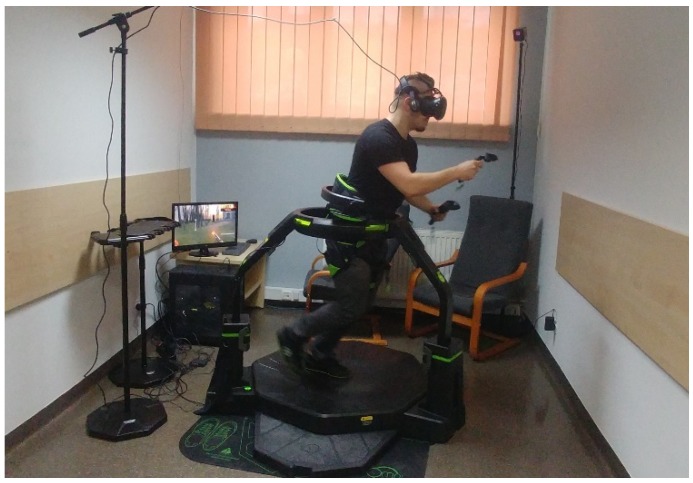
Omni-directional Omni treadmill enabling locomotive movements in virtual reality (VR). Source: author’s elaboration.

**Figure 2:**
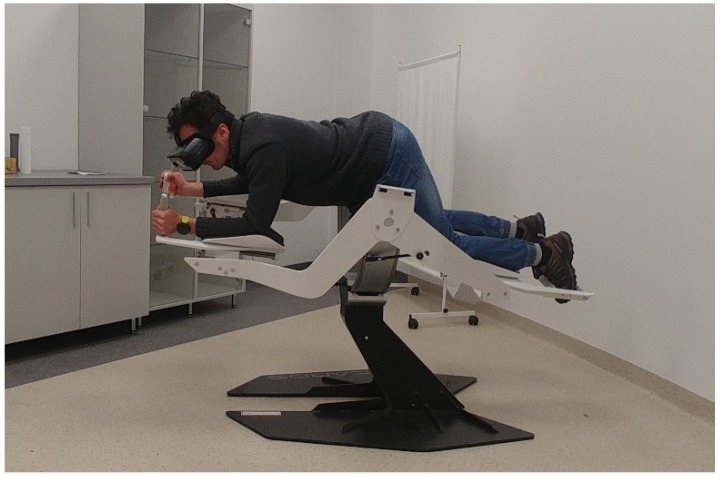
Icaros Pro – flight simulator in VR. Source: author’s elaboration.

The research procedure consisted of a 10-min session of an active video game on at least one of the two studied training devices for PA in IVR (Omni-directional Omni treadmill (Figure 1), Icaros Pro flight simulator (Figure 2)). Before proceeding to the above research activities, the researchers matched the devices to the participant’s height, explained the purpose of the game and explained how to use it and move in VR. Then a 2-min trial game took place, followed by a proper game that lasted 10 min. Users could stop participating in the study at any time. In the group that tested both devices, training sessions were held in the following order: Omni treadmill (O) (dominance of aerobic endurance exercise), Icaros Pro flight simulator (I) (dominance of strength endurance exercise) and a 30-min break to rest between them. 

### 2.3. Methods and Tools

Two AVGs were used to conduct training sessions on the tested devices dedicated to PA in IVR. The game “Travr Training OPS” dedicated to Omni treadmill consists of covering the obstacle course and shooting the indicated targets in the shortest possible time. The necessity to cover the designated course forced the player to perform locomotive movements (walking, running) engaging primarily leg muscles. VR image projection and control of the movement of the upper limbs while moving on the treadmill were carried out thanks to the HTC VIVE goggles (HTC Corporation, New Taipei, Taiwan) and controllers co-operating with the Omni platform. Game ‘Flight’ was used during PA session on the Icaros Pro Flight simulator. The goal of the game was to control a flying ship by moving the body in a position supporting themselves on the forearms and lower legs. The activity was more static than moving on the Omni treadmill, and piloting required the player to constantly control the position of their body and balance in space by appropriate tightening and loosening of the arms, legs and torso muscles. For the projection of the VR image, Samsung Gear goggles (Samsung Electronics Co., Ltd., Suwon, South Korea) were used.

For the assessment of the enjoyment level (EL) Interest/Enjoyment subscale of the Intrinsic Motivation Inventory (IMI) was used [41]. It is a multidimensional measurement grounded on the Self-Determination Theory (SDT) used in assessing the subjective experiences of participants when developing an activity [42]. The Interest/Enjoyment subscale assesses the interest and inherent pleasure when doing a specific activity. It has been used in previous virtual reality exercise studies [8,28,43] and has shown good reliability and validity [41]. According to the inventory, instruction participants ranked their agreement with each statement on a Likert scale of 1 (“not at all true”) to 7 (“very true”). Responses were averaged to create the overall enjoyment scale scores (range 1–7). The subjective assessment of the suitability of PA in VR on the studied training devices was verified using the author’s own questionnaire containing 5 questions with a “yes or no” scale (refer to Table 1 for questions). On the basis of the participants’ answers, fractions of the participants assessing the abovementioned aspects of usefulness PA in VR positively (yes) and negatively (no) were calculated. The questionnaires were completed immediately after the end of the training session. Persons who declared participation in both trials completed the questionnaires after their completion.

During AVGs on the training devices, heart rate (HR) was monitored using the Vantage V pulse meter by Polar (Polar Electro Oy, Kempele, Finland). The intensity of physical exercise was determined on the basis of the average percentage of maximum heart rate (% HRmax) obtained by each participant during the test. Previously, the HRmax value was calculated from the formula by Tanaka et al. (208 − 0.7 × age) [44]. The exercise load was estimated based on the PA intensity classification proposed by the American College of Sport Medicine [45]. According to it, average HR (HRavg) < 64% HRmax means low-intensity, 64% HRmax ≤ HRavg < 77% HRmax—moderate, and HRavg ≥ 77%HRmax—high. The data obtained in this manner was referred to the criteria of health-related recommendations in the scope of intensity of aerobic physical exercises, according to which those of at least moderate intensity (≥64% HRmax) are beneficial for health [46,47]. The total time of HR maintenance during a 10-min effort on the tested training devices was also estimated in three intensity zones: low, moderate and high.

### 2.4. Ethics

The study procedures were reviewed and approved by the Research Ethics Committee of the Jerzy Kukuczka Academy of Physical Education in Katowice. It was conducted in accordance with the Declaration of Helsinki. All participants took part in the study voluntarily and could discontinue their participation at any time. They have provided written consent for the use of information collected during the examination.

### 2.5. Statistical Analysis

Statistica 13.0 (TIBCO Software Inc., Palo Alto, CA, USA) was used to carry out statistical calculations. The analysis of measurement data was carried out using basic descriptive statistics. The results of the survey were presented in percentages. The consistency of the distribution was estimated using the Shapiro-Wilk test. The non-parametric Wilcoxon test was used to assess the significance of the differences between the mean values of results during PA on Omni and Icaros.

## 3. Results

### 3.1. Enjoyment Level

In the group having the Omni training session, the average enjoyment level (EL) during physical activity was 5.74 ± 0.86 points, while in those testing the Icaros flight simulator it was 5.60 ± 0.88 on a 1–7 Likert scale. Comparing the results for excitement/interest subscale of IMI of the participants who completed their training sessions on both training devices demonstrated significant differentiation (*p* < 0.01) of the EL in favor of PA on the first training device (Omni: 6.13 ± 0.90; Icaros: 5.18 ± 0.71 points).

### 3.2. The Intensity Level in the Context of Health Recommendations

**Figure 3:**
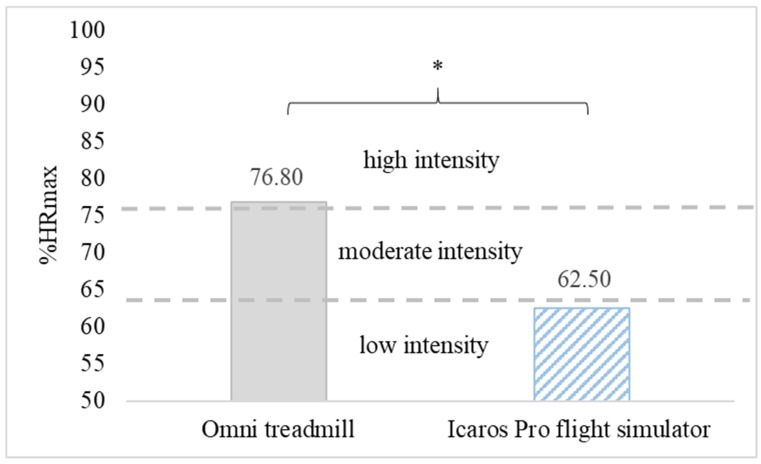
The average intensity of physical effort during active video games (AVGs) in VR on the Omni treadmill and Icaros Pro flight simulator, * *p* < 0.01.

**Figure 4:**
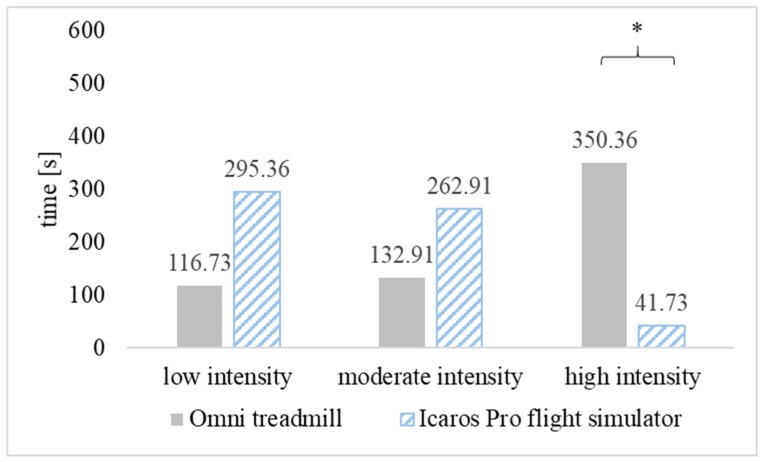
Duration of physical activity at different intensity intervals when practicing 10-min active video games (AVGs) on the Omni treadmill and Icaros Pro flight simulator, * *p* < 0.01.

The average heart rate during PA in VR on the omni-directional Omni treadmill was 149.55 ± 22.31 bpm and was significantly higher (*p* < 0.01) than observed on the Icaros Pro flight simulator—121.36 ± 17.98 bpm. A similar statistically significant relationship was found analyzing the average percentage of maximum heart rate (% HRmax). The estimated parameter for people playing on the treadmill was 76.80% HRmax (high-intensity) and it was significantly higher (*p* < 0.01) than on Icaros Pro—62.50% HRmax (low-intensity) (Figure 3). For the vast majority of time practicing PA in VR on both training devices, the intensity of exercise remained at the level recommended for health benefits (moderate or high). In the case of training on the Omni treadmill, the health-beneficial effort lasted for 80.55% (483.27 s), and on the Icaros Pro flight simulator for 50.77% (304.64 s) of the duration of the game. During Omni training, the high-intensity effort dominated, while practicing AVGs on Icaros was mainly associated with low and moderate physical activity (Figure 4).

### 3.3. The Usefulness of Physical Activity in VR with the Use of Innovative Training Devices in the Opinion of the Participants of the Study

The vast majority of people testing Omni (92%) and Icaros Pro (88%) devices claimed that having this type of training devices they would be practicing PA in VR. Almost every user of the treadmill (97%) and flight simulator (96%) would recommend PA on the tested devices to others.

All the participants were of the opinion that practicing PA on both training devices could be supplementary to PA practiced in free time. A clear majority of users of the multidirectional treadmill (72%) were of the opinion that training in VR on this device can meet the needs of PA practiced in free time in the field of locomotion-based exercises (walking, running). An even more numerous representations of the participants exercising on Icaros (80%) had a similar view on the subject of the tested flight simulator in the context of the possibility of performing exercises in a support position on this training device. A large proportion of people training on the treadmill (44%) were even of the opinion that PA on the Omni platform can replace recreational walking and running. However, most participants testing Icaros (60%) were convinced that simulator training is able to replace typical exercises in a support position (Table 1).

## 4. Discussion

So far, many studies verifying the pro-health nature and enjoyment of physical activity during AVGs have been published [10,48,49,50,51]. Unlike our study, however, they did not evaluate PA in immersive VR, in which man is cut off from the visual and auditory stimuli of the real environment, and instead, he/she receives the image and sound of the simulated world. Moreover, there are few publications aimed at assessing the above-mentioned parameters during PA using innovative VR training devices. In this context, our study seems original.

The assessment of enjoyment during PA is of great importance in shaping the motivational instruction to take action, which from the perspective of public health is currently, among others, regular participation in physical activity [14,52]. The results of many studies have shown that enjoyment is a significant predictor of PA participation regardless of the age of the participants [11,12,13,14] and their health [15,16,17]. Over the past few years, there have been publications on this topic in the context of undertaking physical activity in VR. According to the results of these studies, the attractiveness of various forms of PA in VR is higher in relation to identical forms of PA taken in the classical way [3,18,19].

High enjoyment rating during PA in VR is confirmed by the results of this study. The EL both during physical activity on the Omni treadmill (5.74 ± 0.86 points) as well as on Icaros (5.60 ± 0.88 points) was higher than the level demonstrated by other authors verifying the level of this parameter for various active video games [8,9,10]. A comparison of the enjoyment level during PA between the tested training devices showed a significant difference in favor of the Omni treadmill. The probable reason for this was the need to maintain a proper body balance for continuous 10-min sessions on Icaros, which was a difficult task for the participants. 

In this manuscript, the PA parameters were also evaluated in terms of their health-enhancing character. It turned out that during most of the training sessions in IVR, the intensity of physical activity was on average at the level recommended for health benefits. The pro-health character of active video gaming in VR has also been shown in other studies [33,50,53]. However, in none of them, special devices for PA in IVR were used. Meanwhile, the percentage of population with PA recommended for health by international organizations is low regardless of age or socioeconomic status [54,55,56]. AVGs in IVR helps to eliminate periods of lack of PA, characteristic to traditional computer games, which are one of the most popular leisure-time activities. Therefore, AVGs in IVR can be seen, among others, as tools to fight hypokinesia, especially among children and adolescents and adults up to 35 years of age, who are the main recipients on the computer games market [57].

The high assessment of the usability of PA in VR on training devices is also emphasized by the participants’ declarations, which showed that having such training devices they would be happy to train on them and would recommend this form of PA to their friends. The vast majority of study participants were also of the opinion that PA on the Omni treadmill and the Icaros flight simulator is useful enough to meet the needs of PA practiced in free time, and a large part even claimed they could replace some classic forms of PA with it.

Previously published publications emphasize the potential of PA in IVR considering it a modern trend in healthcare [37], mainly in the field of secondary prevention [18,34,35]. The high assessment of the enjoyment level during physical activity in VR and the conviction of its usefulness as an innovative, attractive form of PA with adequate intensity for health benefits, demonstrated in the study, draw attention to the usefulness of PA in VR on the tested training devices in the context of increasing PA adherence. Such a form of PA, due to its difference from traditional physical effort, can complement the training of regularly physically active people, looking for alternatives to undertaken classical forms of PA, contributing to an increase in their typical PA dose. In addition, the high level of enjoyment associated with physical exertion during IVR seems to be important to overcome insufficient PA frequency often underlined in publications as the main reason for not following pro-health recommendations [58,59,60,61]. In reference to the researches carried out by Banos et al. [18] and, Matsangidou et al. [19], which demonstrated that VR allows for the distraction from the experienced discomfort and lower the perception of pain observed during exercises, PA in IVR on special devices seem to have a potential for the activation of people who avoid PA due to various ailments associated with physical effort.

Taking into account the positive reception of PA in IVR by study participants and the dynamic development of modern computer technology, there are many indications that training devices cooperating with immersive VR can be widely used in the health promotion and physical rehabilitation.

The pilot nature of this manuscript should be emphasized and therefore should be viewed in the context of several limitations. The results of the study concern adults, therefore it would be necessary to verify the other age groups, people with different levels of activity and physical fitness. In the course of further research, it would be recommended to introduce a control group, e.g., performing identical forms of PA, but in a classic way to compare the parameters of such effort with their performance in IVR on the tested devices. In addition, instead of estimating PA intensity on the basis of % HRmax, more objective and standardized methods of measuring it could be used. Based on the obtained results, we can only acknowledge PA in IVR on the tested trainers as having the potential to increase participation in pro-health PA. To verify it, more psychological variables (e.g., Self Determination Theory-related constructs) should be included in the study.

## 5. Conclusions

The results of this study showed that the average enjoyment level during physical activity in VR on the tested training devices was high. In the case of the Omni treadmill, it was 5.74 points, while in the case of the Icaros flight simulator it was 5.60 points. In the opinion of the majority of participants, active video games practiced on the omni-directional Omni treadmill and Icaros flight simulator in immersive VR constitute a useful form of movement to meet the needs of PA practiced in free time, and they can even replace some classic forms of movement.

This study showed the intensity of PA during games on training devices was at the level recommended to obtain pro-health benefits for 80.55% (Omni treadmill) and 50.77% (Icaros Pro flight simulator) of its duration. The average heart rate during activity sessions on the Omni treadmill (149.55 ± 22.31 bpm) was significantly higher than that observed on the Icaros Pro flight simulator (121.36 ± 17.98 bpm), which probably results from a different type of exercise on both training devices. Due to the fact that in the opinion of users, PA in VR on the tested training devices is an enjoyable and useful form of movement, and research shows that its intensity is at the level recommended for obtaining health benefits, it may be assumed that this form of movement can be an effective tool for increasing participation in health-oriented PA.

## Figures and Tables

**Table 1:** Usefulness of physical activity in VR using innovative training devices (omni-directional Omni treadmill and Icaros Pro flight simulator) in the assessment of study participants.

Question	Omni Users (*n* = 36)		Icaros Users (*n* = 25)	
	Yes/ Agree	No/ Disagree	Yes/ Agree	No/ Disagree
If you had an Omni treadmill/Icaros device, would you perform physical activity in VR?	92%	8%	88%	12%
Would you recommend practicing physical activity in VR on the Omni treadmill/Icaros device to others?	97%	3%	96%	4%
Do you think that practicing physical activity on the Omni treadmill/Icaros device can be a supplement to physical activity of an aerobic/strength nature (exercises in a support position) in free time?	100%	0%	100%	0%
Do you think that practicing physical activity on the Omni treadmill/Icaros device can meet the needs related to physical activity performed in free time in the field of locomotion exercises (walking, running) (Omni)/in the area of exercises in a support position (Icaros)?	72%	28%	80%	20%
Do you think that physical activity on the Omni treadmill/Icaros device can replace typical, real forms of physical activity in free time, such as: walking, running (Omni)/typical exercises in a support position (Icaros)?	44%	56%	60%	40%
